# Hydrogen sulfide alleviates high-salt-stimulated myocardial fibrosis through inhibiting hypoxia-inducible factor-1α

**DOI:** 10.3389/fphar.2025.1502269

**Published:** 2025-06-26

**Authors:** Qian Peng, Pan Huang, Boyang Lv, Chaoshu Tang, Hongfang Jin, Yaqian Huang

**Affiliations:** ^1^ Department of Pediatrics, Children’s Medical Center, Peking University First Hospital, Beijing, China; ^2^ Department of Physiology and Pathophysiology, Peking University Health Science Center, Beijing, China; ^3^ State Key Laboratory of Vascular Homeostasis and Remodeling, Peking University, Beijing, China

**Keywords:** hydrogen sulfide, high-salt diet, HIF-1α, myocardial fibrosis, cardiac fibroblasts

## Abstract

**Background:**

Endogenous hydrogen sulfide (H_2_S) and its key generating enzyme, cystathionine β-synthase (CBS), prevent vascular remodeling and damage to target organs during the advancement of hypertension induced by a high-salt diet.

**Objective:**

The contribution of the H_2_S/CBS pathway to high-salt–induced myocardial fibrosis (MF) was explored, with a focus on the mechanistic involvement of hypoxia-inducible factor-1α (HIF-1α).

**Methods:**

We used primary rat cardiac fibroblasts stimulated with high-salt medium and an MF model induced by a high-salt diet in Dahl salt-sensitive rats. Sodium hydrosulfide (NaHS), a commonly used H_2_S donor, was administered *in vitro* at 100 μmol/L and *in vivo* at 90 μmol/kg to maintain adequate H_2_S levels. An HIF-1α stabilizer, dimethyloxalylglycine (DMOG), was used to maintain the HIF-1α protein level. The H_2_S/CBS pathway was followed using Western blotting and a sulfide-sensitive probe. The extent of MF was examined using histological and immunofluorescence staining techniques, including Sirius red and Masson trichrome. We performed Western blot analysis to measure fibrosis-related protein and HIF-1α protein levels.

**Results:**

High-salt exposure reduced H_2_S production and downregulated CBS protein expression in cardiac fibroblasts both *in vitro* and *in vivo*. *In vitro*, the H_2_S donor inhibited the activation of cardiac fibroblasts triggered by high-salt conditions, while *in vivo*, it alleviated MF in salt-sensitive rats. From a mechanistic standpoint, high-salt exposure markedly upregulated HIF-1α expression. However, this increase was reversed by pretreatment with H_2_S. Furthermore, the HIF-1α stabilizer DMOG blocked the H_2_S-induced reduction in HIF-1α protein levels and consequently abolished the antifibrotic effect of H_2_S on cardiac fibroblasts exposed to high-salt conditions.

**Conclusion:**

In conclusion, H_2_S attenuates high-salt-induced MF by suppressing fibroblast activity and collagen synthesis, potentially via downregulation of HIF-1α.

## Graphical Abstract

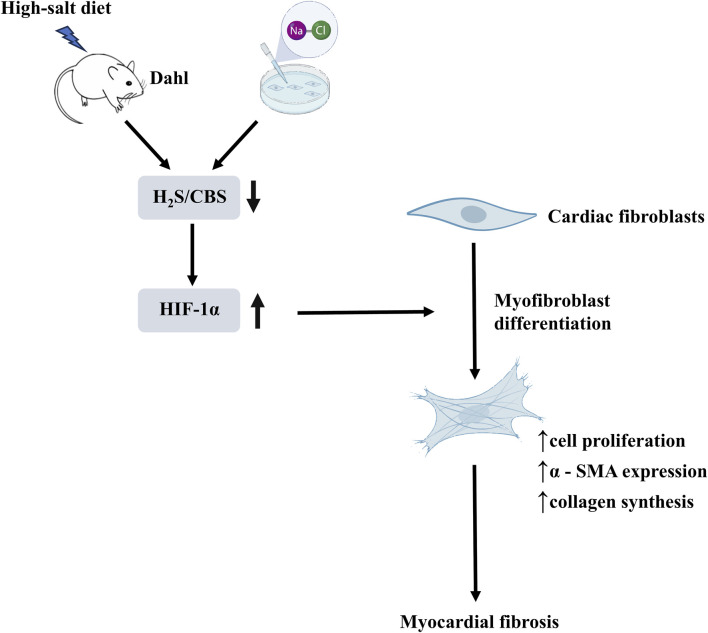

## 1 Introduction

Myocardial fibrosis (MF), a common degenerative condition, occurs during the advanced stages of many cardiovascular diseases. This pathological state is marked by a significant increase in extracellular matrix (ECM) deposition, predominantly involving collagen I and III (González et al., 2018; Frangogiannis, 2019). Excessive ECM deposition can increase the stiffness of the ventricular wall, interfere with myocardial electrical conduction, lead to cardiac dysfunction and arrhythmias, and ultimately cause cardiovascular events including heart failure and infarction-related complications (Bacmeister et al., 2019; Rao et al., 2021). The fibrotic response in the heart is predominantly mediated by activated fibroblasts through enhanced ECM synthesis. Persistent high-salt intake has emerged as a key dietary factor driving myocardial fibrotic changes, potentially mediated by neurohormonal activation, chronic inflammation, and oxidative stress imbalance (Liang and Leenen, 2008; Wu et al., 2016; Esposito et al., 2017). Nevertheless, the exact molecular pathways by which high-salt conditions induce cardiac fibroblast activation and ECM accumulation in MF remain to be fully defined (Zhang et al., 2023).

H_2_S is an endogenously produced gasotransmitter with diverse biological effects (Cao and Bian, 2016). Three enzymes have been identified as key mediators of hydrogen sulfide biosynthesis in mammals, including cystathionine β-synthase (CBS), cystathionine γ-lyase (CSE), and 3-mercaptopyruvate sulfurtransferase (MPST) (Khan et al., 2022). These enzymes exhibit substantial expression in both cardiac and vascular tissues (Geng et al., 2004). H_2_S exerts protective effects across multiple cardiovascular pathologies, such as hypertension, pulmonary hypertension, atherosclerosis, myocardial hypertrophy, and MF (Kang et al., 2020). Simultaneously, H_2_S exerts regulatory effects on various cardiovascular cells (Sun et al., 2019). For example, H_2_S inhibited transforming growth factor-β1-stimulated cardiac fibroblast growth and migration (Zhang et al., 2015). In Dahl rats, excessive salt intake was found to impair H_2_S/CBS pathway function in kidney and myocardial tissues. Conversely, administration of NaHS, a donor of H_2_S, effectively attenuates myocardial hypertrophy, aortic structural remodeling, renal injury, and oxidative stress in cardiac tissue induced by a high-salt diet (Huang et al., 2016; Huang et al., 2017). Evidence supports that the endogenous H_2_S/CBS pathway may exhibit an inhibitory effect against relevant target organ damage resulting from a high-salt diet. However, whether the endogenous H_2_S/CBS system participates in high salt-induced MF, particularly in high salt-induced myocardial fibroblast activation and ECM deposition, and its possible mechanisms remain to be clarified.

HIF-1α functions as a key regulator in cellular adaptation to high-salt conditions (Wang et al., 2010; Ozurumba et al., 2018). Under normoxic conditions, prolyl hydroxylase domain (PHD) enzymes hydroxylate HIF-1α, marking it for degradation via the ubiquitin–proteasome system; in hypoxia, reduced PHD activity permits HIF-1α stabilization and accumulation. Upon stabilization, HIF-1α activates a set of genes involved in metabolic regulation, survival signaling, angiogenesis, and ECM remodeling under hypoxic conditions (Watts and Walmsley, 2019). Under high-salt conditions, HIF-1α activation has been implicated in promoting fibrosis, including kidney fibrosis and vascular remodeling (Della Penna et al., 2014; Deng et al., 2024). In MF, elevated HIF-1α levels contribute to fibrotic development, whereas its inhibition has been shown to alleviate pathological progression (Zou et al., 2022; Niu et al., 2024). Together, these studies suggest that HIF-1α represents a potential therapeutic target in the treatment of fibrosis across multiple organ systems. However, its precise role in high salt-induced MF remains unclear. Emerging evidence suggests H_2_S may regulate HIF-1α under certain conditions. H_2_S suppresses HIF-1α protein expression through multiple mechanisms, including inhibition of the PHD2/HIF-1α/MAPK signaling cascade, persulfidation of PHD2, and modulation of translational regulation (Wu et al., 2012; Dey et al., 2020; Guan et al., 2020). However, it is not fully established whether the endogenous H_2_S/CBS pathway mitigates high-salt-induced MF by inhibiting HIF-1α.

In this study, a MF model was established in Dahl rats by chronic high-salt feeding, and an *in vitro* system using primary cardiac fibroblasts treated with high-salt medium was employed to assess fibroblast responses. These models were used to examine alterations in the endogenous H_2_S/CBS pathway and its contribution to MF progression. To elucidate the underlying mechanism, HIF-1α expression was modulated using the stabilizer DMOG to assess its involvement in cardiac fibroblast activation and ECM deposition.

## 2 Materials and methods

### 2.1 Reagents

Beijing Keao Xieli Feed Company (Beijing, China) supplied the high-salt diet containing 8% sodium chloride (NaCl). The HIF-1a stabilizer DMOG (S7483, Selleck, TX, United States), which is a cell-permeable inhibitor that competes with HIF-hydroxylated prolyl-hydroxylase (Nowak-Stępniowska et al., 2022), was used in this study. NaHS (161527, Sigma-Aldrich, MO, United States) and NaCl (S8211, Solarbio, Beijing, China) were used in this study. The primary antibodies included CBS (sc-67154, Santa Cruz, Dallas, TX, United States), CSE (12217-1-AP, Proteintech, Wuhan, China), MPST (sc-376168, Santa Cruz, Dallas, TX, United States), HIF-1α (ab179483, Abcam, Cambridge, United Kingdom), collagen I (A21059, ABclonal, Wuhan, China), collagen III (ab184993, Abcam, Cambridge, United Kingdom), PCNA (10205-2-AP, Proteintech, Wuhan, China), α-SMA (ab124964, Abcam, Cambridge, United Kingdom), β-tubulin (TA-10, Zhongshan Golden Bridge, Beijing, China), β-actin (20536-1-AP, Proteintech, Wuhan, China), and GAPDH (TA-08, Zhongshan Golden Bridge, Beijing, China). The main reagents used for cell culture were FBS (FB25015, CLARK Bioscience, United States), 100 × PS (15140-122, Thermo Scientific, United States), 100 × glutathione (LG) (25030081, Thermo Scientific, United States), and trypsin containing EDTA (0.25%) (25200-056, Thermo Scientific, United States).

### 2.2 Animal model establishment

Animal experiments were conducted following approval from the Animal Research Ethical Committee of Peking University First Hospital (J201205) and in compliance with relevant institutional ethical standards.

Thirty male salt-sensitive Dahl rats (6 weeks old) were sourced from Charles River Laboratories (Beijing, China; License No. SCXK 2012-0001) and housed at the Animal Experimental Center of Peking University First Hospital. Three experimental groups were formed by randomly distributing 10 rats into each group: a control group, which received 0.5% NaCl chow and daily intraperitoneal injections of 0.9% saline; a high-salt group, which was fed 8% NaCl chow along with saline injections; and a high-salt + NaHS group, which received 8% NaCl chow combined with daily intraperitoneal administration of NaHS at 90 μmol/kg. NaHS was freshly dissolved each day in 0.9% saline and administered for 8 consecutive weeks. Both the control and high-salt groups received equal volumes of saline via daily intraperitoneal injection for the same duration. Following 8 weeks of intervention, rats received 12% urethane (10 mL/kg, i. p.) for anesthesia, and hearts were promptly collected for further analysis.

### 2.3 Histology

Collagen accumulation in myocardial tissue was assessed by Masson trichrome staining and Sirius red staining. Cardiac tissues were excised and rinsed thoroughly with ice-cold PBS. Subsequently, a solution containing 4% paraformaldehyde was used to fix the tissue, followed by paraffin wax to encase the tissue and divide it into consecutive sections (4 μm). Masson trichrome staining (#G1346, Solarbio, Beijing, China) and Sirius Red staining (BP-DL030, Solarbio, Nanjing, China) were carried out following the manufacturer’s protocols. To analyze fibrosis, six non-overlapping fields (400×) were randomly selected from each slice. Fibrotic and total tissue areas were measured with ImageJ (NIH, Bethesda, MD, United States).

### 2.4 *In Vitro* experiments

Hearts were excised from male Sprague–Dawley rats (80–100 g) for the isolation of cardiac fibroblasts. After euthanasia by CO_2_ asphyxiation, a 1 min immersion in 75% ethanol was performed to sterilize the external surface. The hearts were removed under aseptic conditions and washed in pre-chilled PBS with 1% penicillin/streptomycin to remove residual blood. Cardiac tissue was sectioned into approximately 1 mm^3^ blocks and placed in T25 flasks to facilitate attachment through static incubation. Next, 20 mL of Dulbecco Modified Eagle Medium/Nutrient Mixture F-12 (DMEM/F-12) containing 20% fetal bovine serum (FBS) and 1% penicillin/streptomycin was added to the culture flask. Cell cultures were incubated at 37°C with 5% CO_2_ under humidified conditions. Upon reaching 70%–80% confluency, they were digested with 0.25% trypsin and resuspended in complete DMEM/F-12 supplemented with 10% FBS.

The sodium concentration in the DMEM/F-12 medium was 137 ± 1.0 mmol/L. To obtain a high-salt medium, an appropriate amount of NaCl was added to the DMEM/F-12 medium such that the final sodium concentration in the high-salt medium reached 161 mmol/L. After seeding primary cardiac fibroblasts (1 × 10^5^ cells/well) into six-well plates, cells were maintained at 37°C with 5% CO_2_ for 24 h prior to experimental treatment. After adhesion, cells were treated with high-salt medium (161 mmol/L Na^+^) for 48 h, following a previously validated protocol for inducing fibrotic responses in cardiac fibroblasts (Liu et al., 2020). Cells in the control group were exposed to standard DMEM/F-12 for 48 h. In the high-salt + NaHS group, the H_2_S donor NaHS (100 μmol/L) was administered for three distinct durations (12, 24, or 48 h) to assess the temporal effects of H_2_S treatment under high-salt conditions. In some experiments, cells were subjected to a pre-treatment with 20 μmol/L DMOG for 6–8 h (Xu et al., 2022).

NIH3T3 cells (mouse embryonic fibroblasts), obtained from the Cell Bank of the Chinese Academy of Sciences (Beijing, China), were cultured in DMEM with 10% FBS at 37°C under 5% CO_2_ and humidified conditions. When cell confluence approached 90%, they were detached using 0.25% trypsin for 3–5 min and transferred to six-well culture plates at 1 × 10^4^ cells/cm^2^. Following a 24-h incubation period, cells were shifted to DMEM with 1% FBS to initiate serum starvation and kept in this low-serum environment for another 12–16 h. Cells were subsequently exposed for 48 h to medium containing either physiological or high sodium concentrations (161 mM NaCl). An H_2_S donor (NaHS, 100 μmol/L) was used to provide sufficient H_2_S levels.

Cardiomyocytes were harvested from neonatal Sprague–Dawley rats (2–3 days old) using the Pierce™ Primary Cardiomyocyte Isolation Kit (#88281Y, Thermo Scientific, United States), in accordance with the manufacturer’s instructions. After mincing, left ventricular tissue was digested at 37°C for 30 min in 200 μL of an enzyme mix (Enzymes 1 and 2) provided in the cardiomyocyte isolation kit. The enzymatic digestion was terminated by adding 1 mL of DMEM containing 10% FBS, followed by gentle pipetting to obtain a single-cell suspension. The suspension was then transferred to a 6-well plate for subsequent treatments. Cells were exposed to either standard or high-sodium medium (161 mM NaCl) for 48 h. To ensure adequate H_2_S levels, an H_2_S donor (NaHS, 100 μmol/L) was supplemented into the culture medium.

### 2.5 Measurement of H_2_S production

The concentration of H_2_S in the culture supernatant was quantified using a TBR4100 free radical detection system (World Precision Instruments, Shanghai, China) (Fox et al., 2012). Initially, 5× PBS buffer with a pH of 7.2 and a concentration of 50 mmol/L was used to establish a consistent reference current (typically ranging from 100 to 2000 pA). Once a stable current was achieved, a calibration curve was generated using PBS-diluted sodium sulfide (0.5–16 μmol/L) for quantifying H_2_S in the collected samples. The probe into each sample had an immersion depth of approximately 10–15 mm. H_2_S concentrations in the samples were quantified using the standard curve.

### 2.6 Immunofluorescence staining

After an initial rinse with PBS, fixation was performed using paraformaldehyde at 4% for 20 min, followed by a 10-min permeabilization step in PBS containing 0.3% Triton X-100. To minimize non-specific interactions, samples were incubated with 5% BSA for 1 h. Each of these preparatory steps was conducted under ambient conditions. Subsequently, cells were incubated overnight at 4°C with the appropriate primary antibodies (1:100 for collagen I and III, 1:2000 for α-SMA). The next day, cells were incubated at room temperature for 1 h with a secondary antibody (Invitrogen, Carlsbad, CA, United States). Nuclear staining was achieved using DAPI, followed by confocal imaging (Leica Microsystems) to detect fluorescence.

### 2.7 Western blot

Protein extraction from cardiac tissue and cell samples was performed using RIPA buffer (P0013B; Beyotime, Shanghai, China) according to standard protocols. Supernatants obtained after centrifugation were subjected to protein quantification via a BCA assay (P0011-1; Beyotime, Shanghai, China). Protein samples were prepared by mixing lysates with 2× loading buffer and boiling for 10 min. We resolved proteins using 10% SDS–PAGE, followed by transfer onto nitrocellulose membranes. Fat-free milk with a concentration of 5% was utilized for a blocking process lasting 60 min at a temperature of 37°C. Membranes were exposed to primary antibodies (4°C, overnight) at the following ratios: 1:1,000 for CBS, CSE, MPST, HIF-1α, collagen I, and collagen III; 1:10,000 for PCNA; 1:20,000 for α-SMA; 1:2000 for β-tubulin; and 1:5,000 for GAPDH and β-actin. On the next day, membranes underwent three PBST washes, followed by a 1-h incubation with species-specific secondary antibodies at 37°C. After ECL reagent application, signal development was performed using a gel imaging system (Protein Simple, United States).

### 2.8 Real-time quantitative polymerase chain reaction

Cultured cells were lysed using TRIzol reagent (Cat# 15596026, Invitrogen, United States) for total RNA isolation, following standard protocols. The integrity and yield of the RNA were evaluated via spectrophotometric analysis using a NanoDrop 2000 system (Thermo Scientific, United States). Complementary DNA was generated using oligo (dT) primers (Cat# C110A; Promega, United States) and an M-MLV enzyme (M5313; Promega, United States). qPCR assays were then conducted using the GoTaq Master Mix (Promega, A600A, United States) on a 7500-detection platform (Applied Biosystems, United States), with fluorescence data collected throughout the amplification cycles. Transcript levels were analyzed using the 2^−ΔΔCt^ method and normalized against β-actin. The primer sets are shown in Supplementary Table S1.

### 2.9 Statistical analysis

All statistical analyses were conducted using GraphPad Prism 10.0 (GraphPad Software, CA, United States). All values are shown as mean ± SD. For two-group comparisons, an unpaired Student’s t-test was utilized, while group differences among more than two conditions were analyzed via ANOVA. Statistical significance was defined as p < 0.05.

## 3 Results

### 3.1 High-salt stimulation downregulates the H_2_S/CBS pathway in cardiac fibroblasts

**FIGURE 1:**
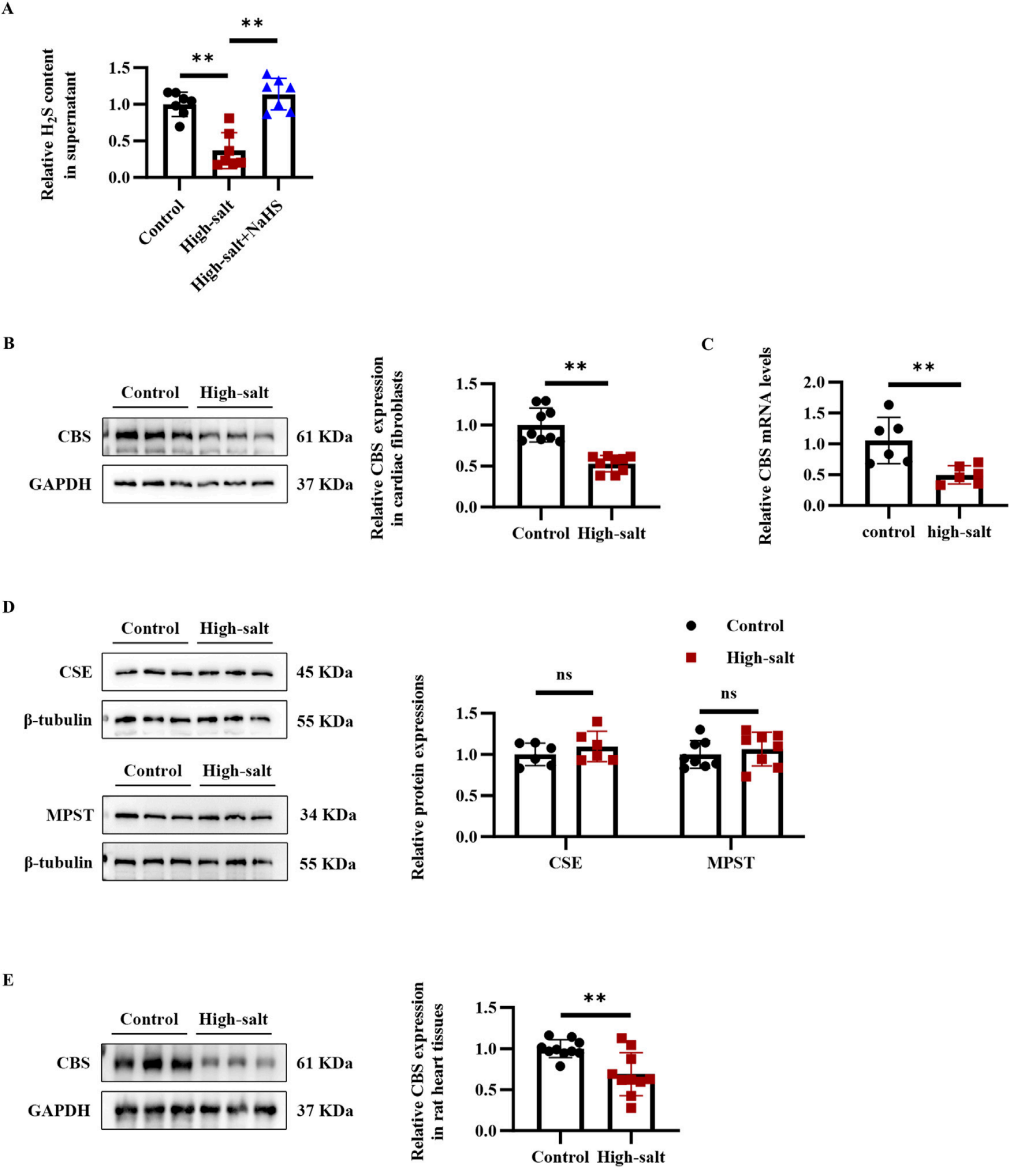
High-salt stimulation downregulated the endogenous H_2_S/CBS pathway in cardiac fibroblasts *in vitro* and in rat myocardial tissues *in vivo*. **(A)** H_2_S concentration in the supernatant of cardiac fibroblasts detected by the free radical analyzer TBR4100 (n = 7 per group). **(B)** Western blot analysis of CBS protein expression in cardiac fibroblasts (n = 9 per group). **(C)** qRT-PCR analysis of CBS mRNA levels in cardiac fibroblasts (n = 9 per group). **(D)** Western blot analysis of CSE and MPST protein expression in cardiac fibroblasts (n = 6 per group). **(E)** Western blot analysis of CBS protein expression in the myocardial tissues of Dahl rats (n = 10 per group). Results are expressed as mean ± SD. *P < 0.05; **P < 0.01; ns, not significant. H_2_S, hydrogen sulfide; CBS, cystathionine β-synthase; CSE, cystathionine γ-lyase; MPST, mercaptopyruvate sulfurtransferase.

To investigate changes in the endogenous H_2_S/CBS pathway during high-salt-induced cardiac fibrosis, both Dahl rats and cardiac fibroblasts were exposed to elevated salt conditions. Following 48 h of high-salt exposure, cardiac fibroblasts exhibited markedly lower levels of H_2_S, along with decreased CBS mRNA and protein expression, relative to untreated controls (Figures 1A–C). In contrast, CSE and MPST protein levels showed no detectable change in cardiac fibroblasts exposed to high-salt conditions (Figure 1D). In addition, after treatment with a high-salt diet, Dahl rats exhibited reduced CBS protein expression in the myocardial tissues (Figure 1E). Collectively, the data demonstrate that high-salt conditions inhibit endogenous H_2_S generation and suppress CBS expression in cardiac fibroblasts, both *in vitro* and *in vivo*.

### 3.2 H_2_S attenuates high-salt-induced myocardial fibrosis in dahl rats

**FIGURE 2:**
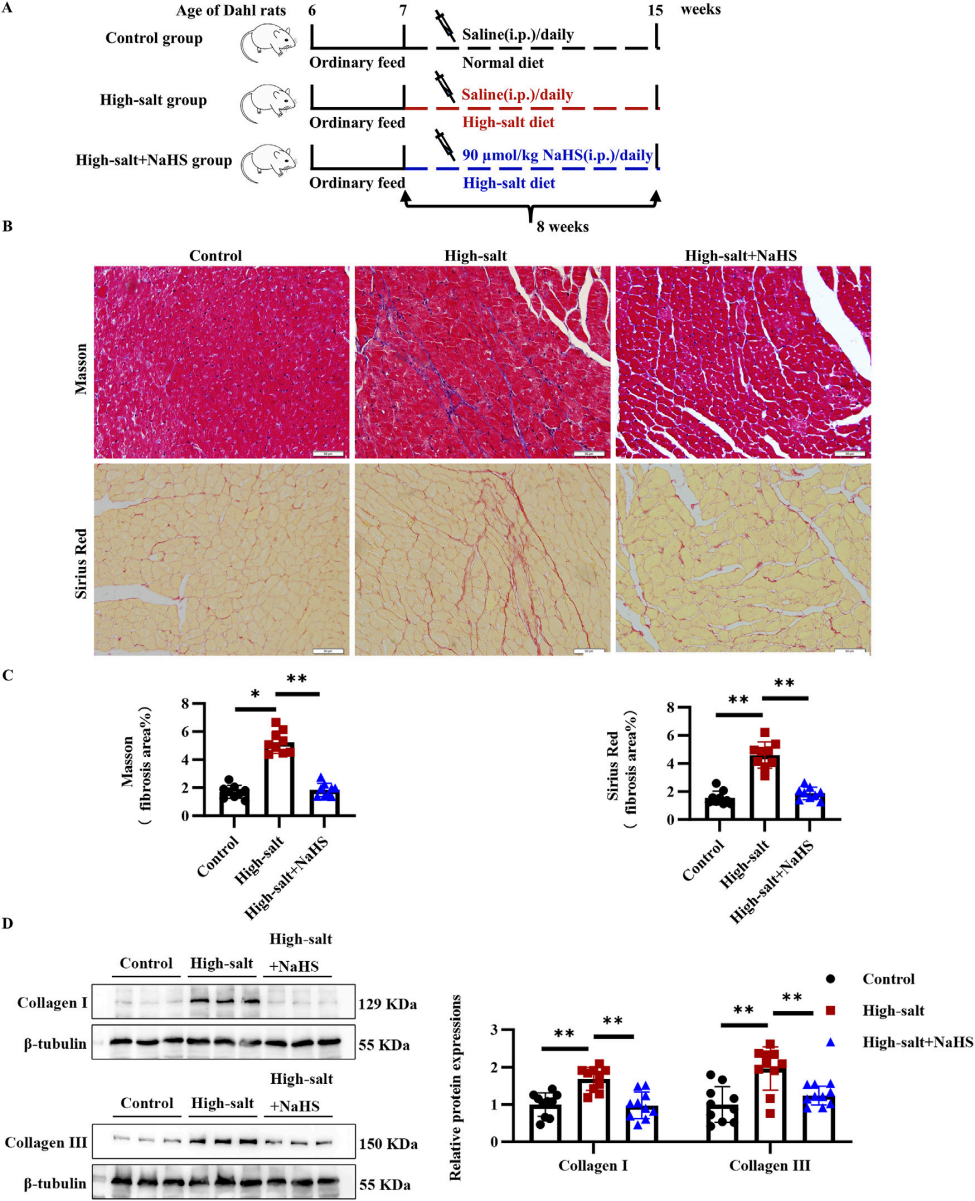
H_2_S significantly attenuated myocardial fibrosis in Dahl rats fed with a high-salt diet *in vivo*. **(A)** Schematic representation of the design of the animal experimental process, including the intraperitoneal injection of NaHS and different dietary interventions. **(B)** Representative Masson trichrome and Sirius red staining. In the Masson trichrome staining, the collagen area is blue, and the collagen area is red in the Sirius red staining. **(C)** Quantification of Masson trichrome and Sirius red staining (n = 9 per group). Scale bar = 50 μm. **(D)** Western blot analysis of collagens I and III in the myocardial tissues of Dahl rats (n = 10 per group). Results are expressed as mean ± SD. *P < 0.05; **P < 0.01.

To elucidate the contribution of H_2_S/CBS pathway suppression to MF progression, we administered daily intraperitoneal injections of the H_2_S donor NaHS to high salt-fed Dahl rats (Figure 2A). High-salt intake markedly increased cardiac interstitial fibrosis, as shown by Masson trichrome and Sirius red staining (Figures 2B,C). Western blotting further confirmed elevated collagen I and III levels in the myocardium of rats receiving high-salt treatment (Figure 2D). In contrast, NaHS administration effectively reduced interstitial fibrosis and downregulated myocardial collagen I and III expression in the high-salt group (Figures 2B–D). These findings suggest that H_2_S significantly attenuates high-salt-induced MF *in vivo*.

### 3.3 H_2_S attenuates high-salt-induced cardiac fibroblast activation and collagen deposition by inhibiting HIF-1α

**FIGURE 3:**
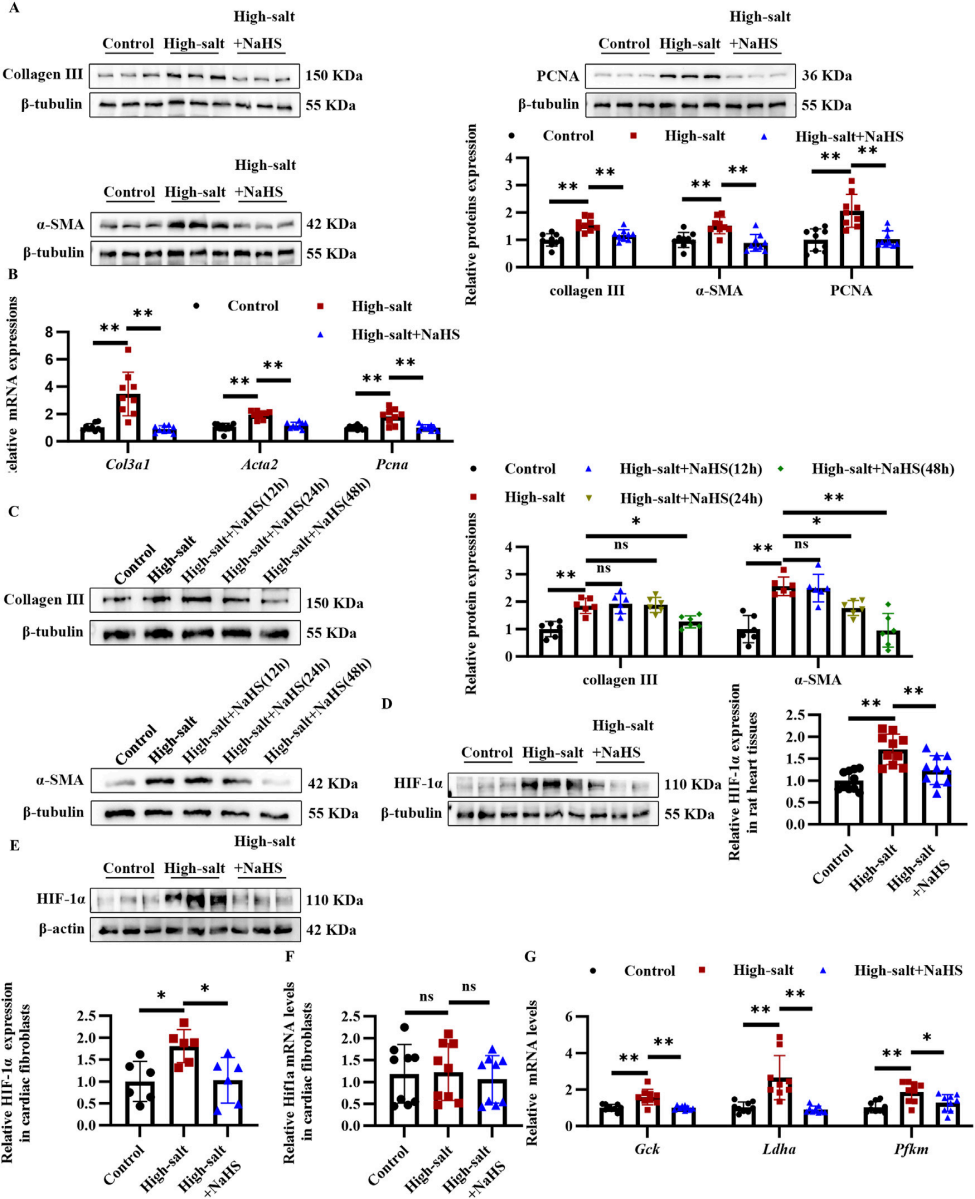
H_2_S attenuates high salt-induced cardiac fibroblast activation and collagen deposition via suppression of HIF-1α. **(A)** Western blot analysis of collagen III, α-SMA, and PCNA protein expression (n = 9 per group). **(B)** qRT-PCR analysis of *Col3a1*, *Acta2*, and *Pcna* mRNA levels in cardiac fibroblasts (n = 9 per group). **(C)** Western blot analysis of collagen III and α-SMA protein expression in cardiac fibroblasts treated with NaHS for 12 h, 24 h, and 48 h (n = 6 per group). **(D)** Western blot analysis of HIF-1α protein level in the myocardial tissues of Dahl rats (n = 10 per group). **(E,F)** Protein **(E)** and mRNA **(F)** expression of HIF-1α in cardiac fibroblasts (n = 6 per group). **(G)** qRT-PCR analysis of HIF1α-targeted key glycolytic enzyme genes in cardiac fibroblasts (n = 9 per group). Results are expressed as mean ± SD. *P < 0.05; **P < 0.01; ns, not significant. HIF-1α, hypoxia-inducible factor-1α; H_2_S, hydrogen sulfide.

To further examine the influence of H_2_S on cardiac fibroblast activation and collagen synthesis, primary rat cardiac fibroblasts were exposed to a high-salt medium and the H_2_S donor, NaHS, for 48 h. Under high-salt conditions, collagen III, α-SMA, and PCNA protein levels were markedly increased, as shown by Western blot analysis (Figure 3A). However, NaHS pre-treatment effectively restored H_2_S levels in the culture supernatant and markedly attenuated these pathological changes (Figures 1A, 3A). Consistently, RT-qPCR results demonstrated that NaHS suppressed the high-salt-induced transcriptional upregulation of fibrogenic genes, including *Col3a1, Acta2,* and *Pcna* (Figure 3B). Notably, H_2_S treatment for 48 h, but not 12 or 24 h, nearly fully reversed the high-salt-induced fibrotic phenotypes, prompting its use in subsequent experiments (Figure 3C). Collectively, these findings indicate that NaHS effectively mitigates high-salt-triggered activation, proliferation, and ECM synthesis in cardiac fibroblasts.

To explore how H_2_S exerts its antifibrotic effects at the molecular level, we investigated HIF-1α, a well-established regulator of fibrotic processes under high-salt conditions. We postulated that HIF-1α may mediate the antifibrotic effect of H_2_S under high-salt conditions. To investigate this hypothesis, we first examined HIF-1α protein expression in both Dahl rat models and cardiac fibroblasts exposed to high-salt conditions, with or without H_2_S donor treatment. Myocardial HIF-1α expression was significantly increased in high-salt-fed rats, whereas NaHS treatment substantially reduced its levels, as shown by Western blot analysis (Figure 3D). Consistent with the *in vivo* results, the cell-based experiments demonstrated that NaHS effectively suppressed high-salt-induced upregulation of HIF-1α in cardiac fibroblasts (Figure 3E). The RT-qPCR analysis confirmed that *Hif1a* mRNA expression remained unchanged in cardiac fibroblasts under these conditions (Figure 3F). In line with previous studies, H_2_S did not affect *Hif1a* mRNA levels, indicating no change at the transcriptional level (Wu et al., 2012). At the downstream level, the HIF-1α target genes—glucokinase (*Gck*), lactate dehydrogenase A (*Ldha*), and muscle phosphofructo-1-kinase (*Pfkm*)—were transcriptionally upregulated in cardiac fibroblasts upon high-salt exposure (Figure 3G). Notably, NaHS treatment not only suppressed HIF-1α protein expression but also reduced mRNA levels of its target genes (Figure 3G). These findings indicate that H_2_S suppresses HIF-1α and its downstream targets in cardiac fibroblasts and Dahl rats exposed to high salt, potentially contributing to its anti-fibrotic effects.

### 3.4 HIF-1α mediates the antifibrotic effects of H_2_S in high-salt-stimulated cardiac fibroblasts

**FIGURE 4:**
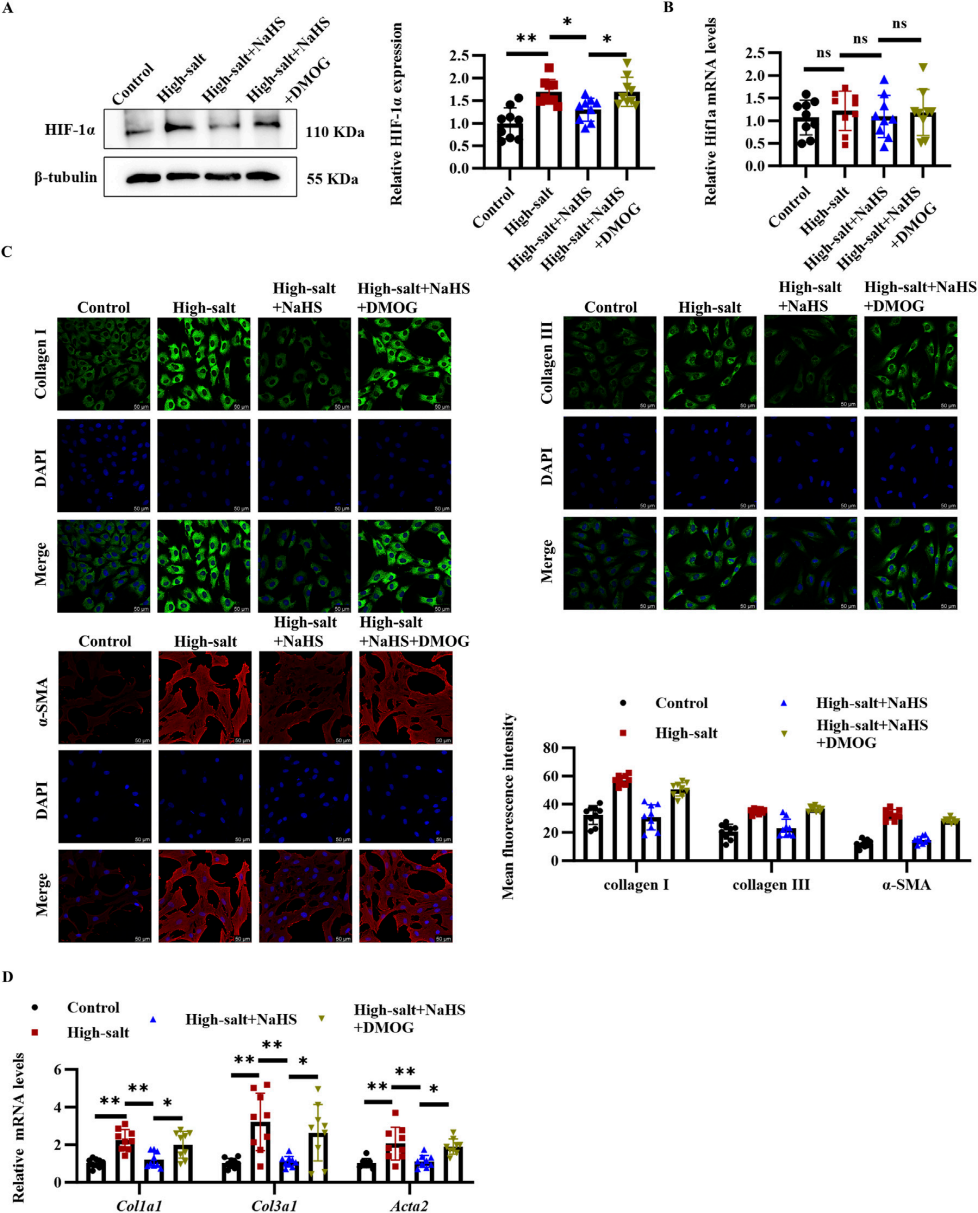
DMOG upregulated HIF-1α protein level, and reversed the inhibitory effect of H_2_S on cardiac fibroblast activation and collagen deposition. **(A,B)** Protein **(A)** and mRNA **(B)** expression of HIF-1α in cardiac fibroblasts (n = 9 per group). **(C)** Representative immunofluorescence images and quantification of collagen I, collagen III, and α-SMA protein levels in cardiac fibroblasts; n = 9; Scale bar = 50 μm. **(D)** qRT-PCR analysis of *Col1a1*, *Col3a1*, and *Acta2* mRNA levels in cardiac fibroblasts (n = 9 per group). Results are expressed as mean ± SD. *P < 0.05; **P < 0.01; ns, not significant. DMOG, dimethyloxalylglycine; HIF-1α, hypoxia-inducible factor-1α.

To determine whether HIF-1α acts as a critical mediator in the antifibrotic actions of H_2_S, we employed the HIF-1α stabilizer DMOG to maintain HIF-1α protein levels in cardiac fibroblasts exposed to high-salt and NaHS conditions. Pre-treatment with DMOG increased the HIF-1α protein levels, rather than mRNA levels, in the cardiac fibroblasts exposed to the high-salt and NaHS (Figures 4A,B). Importantly, this HIF-1α stabilization effectively reversed the antifibrotic effects of NaHS, as evidenced by immunofluorescence staining revealing restored expression of collagen I, collagen III, and α-SMA in cardiac fibroblasts from the high-salt + NaHS + DMOG group compared with the high-salt + NaHS group (Figure 4C). Consistent with these findings, the RT-qPCR analysis demonstrated that DMOG treatment markedly increased the mRNA levels of fibrogenic genes *Col1a1*, *Col3a1*, and *Acta2* in the high-salt + NaHS + DMOG group compared to the high-salt + NaHS group (Figure 4D). Collectively, these findings suggest that the antifibrotic effects of H_2_S are mediated through the HIF-1α pathway by regulating cardiac fibroblast activation and ECM deposition.

### 3.5 H_2_S alleviates the high-salt-induced HIF-1α protein level and collagen deposition in NIH3T3 fibroblasts and primary cardiomyocytes

**FIGURE 5:**
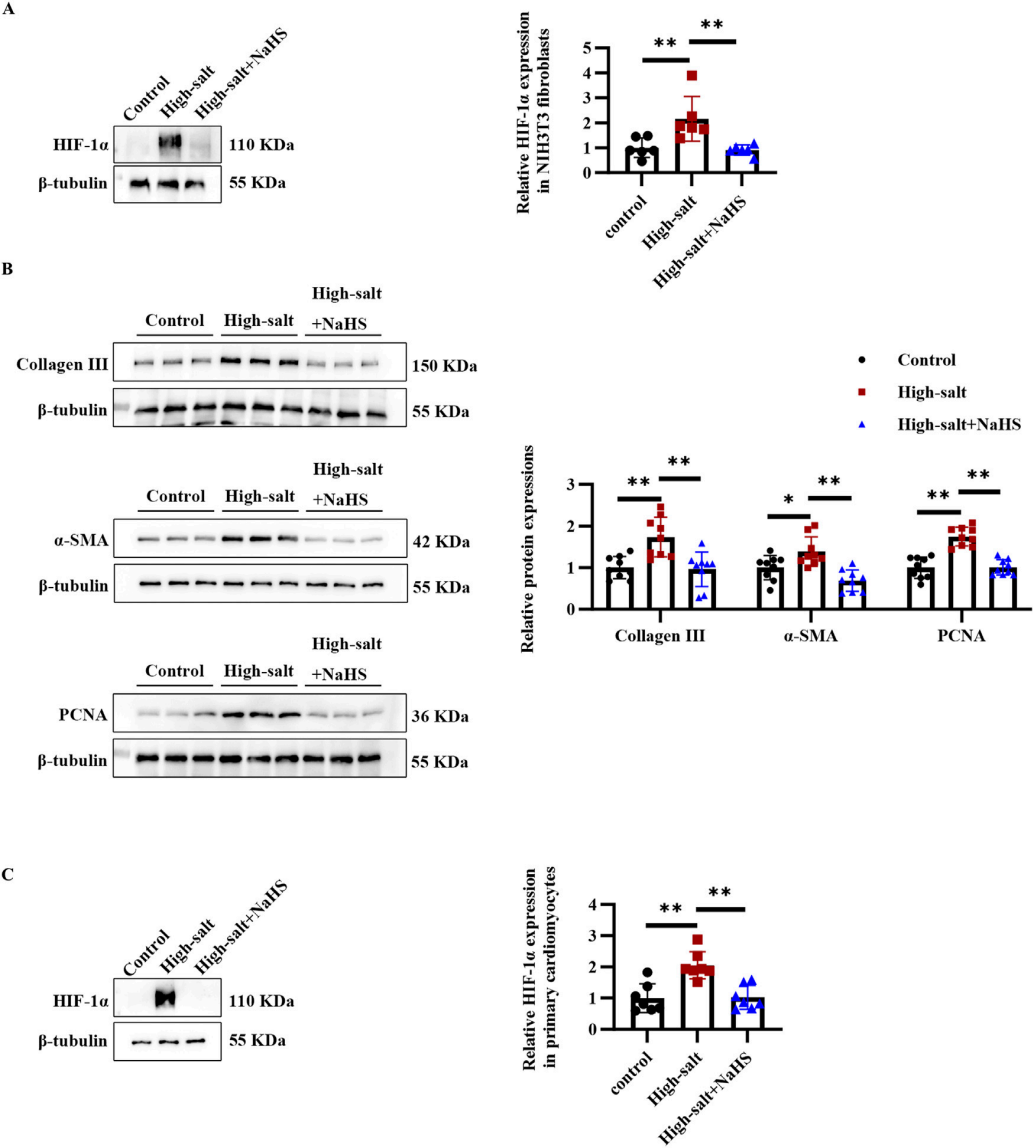
H_2_S alleviated high salt-induced HIF-1α protein level and collagen deposition in NIH3T3 fibroblasts and primary cardiomyocytes. **(A)** Western blot analysis of HIF-1α protein level in NIH3T3 fibroblasts (n = 6 per group). **(B)** Western blot analysis of collagen III, α-SMA, and PCNA protein expression in NIH3T3 fibroblasts (n = 9 per group). **(C)** Western blot analysis of HIF-1α protein level in primary cardiomyocytes (n = 6 per group). Results are expressed as mean ± SD. *P < 0.05; **P < 0.01. HIF-1α, hypoxia-inducible factor-1α.

Given that high-salt stimulation induced HIF-1α upregulation and fibrosis-related changes in rat primary cardiac fibroblasts, we sought to determine whether these effects were consistent across different cell types. To this end, we extended our investigation to NIH3T3 fibroblasts, a widely used cell line for studying fibrosis and primary cardiomyocytes. In NIH3T3 fibroblasts, high-salt exposure significantly upregulated HIF-1α and fibrosis-related markers, including collagen III, α-SMA, and PCNA (Figures 5A,B). Notably, NaHS treatment effectively inhibited high-salt-induced upregulation of HIF-1α, collagen III, α-SMA, and PCNA protein in NIH3T3 fibroblasts (Figures 5A,B). In addition, the Western blot analysis revealed that high-salt stimulation significantly upregulated HIF-1α expression in primary cardiomyocytes (Figure 5C). In contrast, NaHS treatment markedly suppressed high-salt-induced HIF-1α upregulation in primary cardiomyocytes (Figure 5C). These findings are consistent with those from primary cardiac fibroblasts and further highlight HIF-1α as a critical mediator of H_2_S-induced protection against high-salt-induced fibrosis.

## 4 Discussion

**FIGURE 6:**
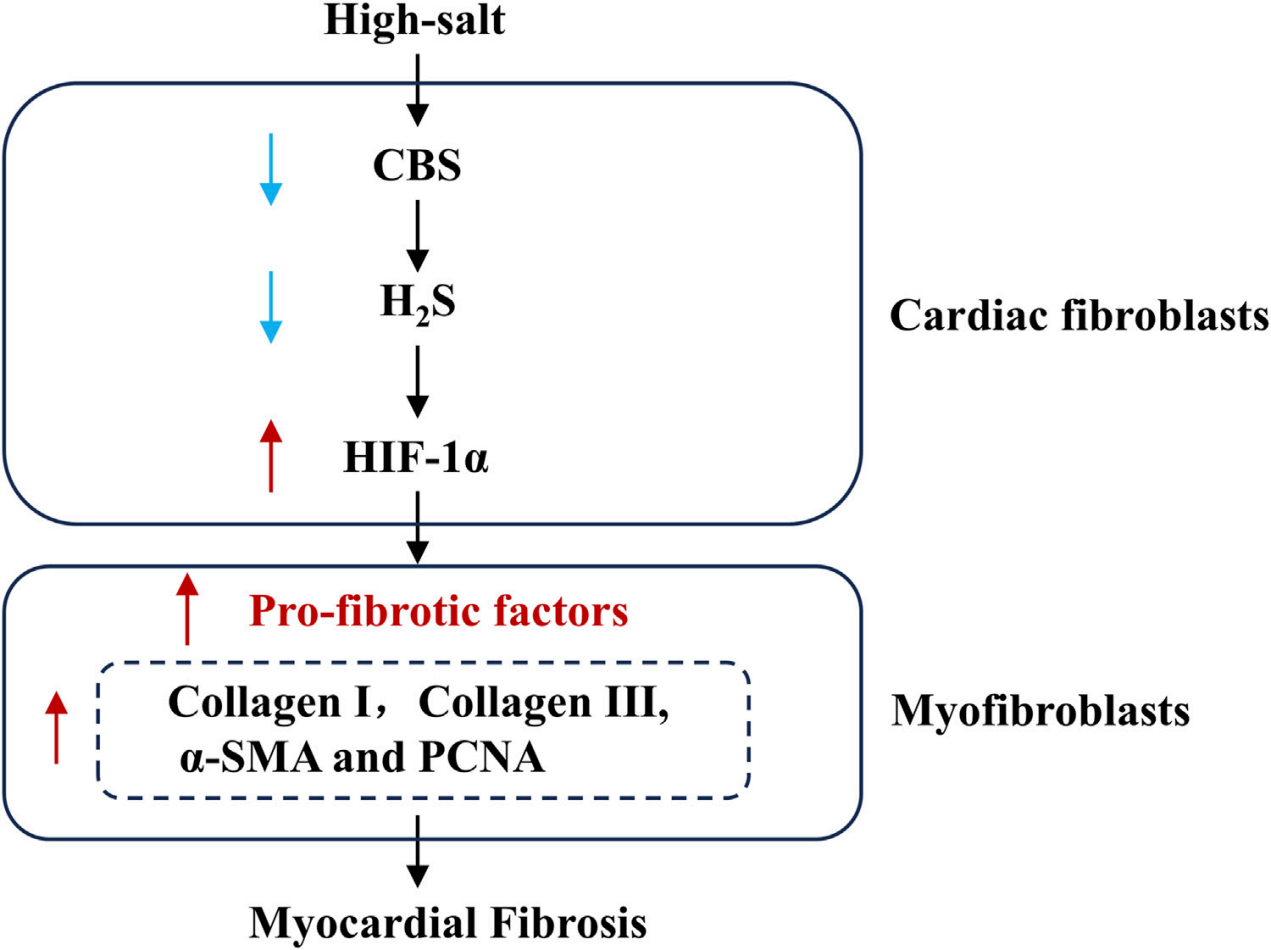
Schematic diagram. Downregulation of the endogenous H_2_S/CBS pathway is an important mechanism underlying high salt-induced MF. High salt downregulates CBS protein expression, subsequently decreasing myocardial H_2_S generation, facilitating HIF-1α protein expression, promoting cardiac fibroblast activation and collagen deposition, and ultimately leading to MF. H_2_S, hydrogen sulfide; CBS, cystathionine β-synthase.

High-salt stimulation promoted the progression of MF, which was accompanied by a reduction in H_2_S levels and reduced expression of CBS in cardiac fibroblasts. Notably, exposure to H_2_S mitigated high-salt-triggered fibroblast activation and subsequent myocardial fibrogenesis. Mechanistically, the antifibrotic effect of H_2_S under high-salt conditions may involve the downregulation of HIF-1α expression in cardiac fibroblasts (Figure 6). Our findings reveal a distinct molecular mechanism through which high-salt exposure contributes to MF pathogenesis.

Chronic high-salt consumption has emerged as a significant driver of cardiovascular disease worldwide (Ma et al., 2022). Although salt intake has been recommended to be reduced since more than 20 years, it remains steadily high (Little and Ellison, 2024). A deeper understanding of the molecular pathways driving high-salt-related cardiovascular injury is essential for developing effective interventions that reduce health risks without compromising dietary preferences. Therefore, a high-salt diet-induced animal model was established in this study. H_2_S is a critical gaseous signaling molecule that protects against cardiovascular disorders (Kolluru et al., 2023). Our earlier research identified a selective downregulation of the H_2_S/CBS pathway in the heart and kidneys of Dahl rats after prolonged high-salt intake, while leaving other H_2_S-producing enzymes (CSE and MPST) unaffected (Huang et al., 2016; Huang et al., 2017). The specificity of this downregulation was further confirmed in the present study. High-salt stimulation significantly suppressed CBS protein and mRNA expression in cardiac fibroblasts, without affecting CSE and MPST. Notably, supplementation with the H_2_S donor NaHS mitigated high-salt-induced pathologies, including aortic structure remodeling, kidney injury, excessive myocardial oxidative stress, and cardiac hypertrophy (Huang et al., 2016; Huang et al., 2017). While the H_2_S/CBS pathway has demonstrated protective effects against high-salt-induced organ damage, the mechanisms underlying its involvement in high-salt-mediated MF still require further investigation.

High-salt conditions have been shown to elicit fibrotic responses across diverse *in vivo* and *in vitro* models. In animal experiments, high-salt diet-fed male Wistar rats developed MF regardless of their blood pressure levels. Additionally, under high-salt conditions, cardiac fibroblasts exhibited enhanced proliferation and collagen synthesis (Ferreira et al., 2010; Liu et al., 2020). Interestingly, NaHS treatment attenuated MF in Dahl rats fed a high-salt diet, and concurrently reduced fibroblast activation and ECM accumulation in cardiac fibroblasts exposed to high-salt stimulation. These data indicate that a decrease in the endogenous H_2_S/CBS pathway could contribute to MF induced by high-salt stimulation. NaHS was delivered by daily intraperitoneal injection at a dosage of 90 μmol/kg throughout the study period. Intraperitoneal administration yields superior bioavailability relative to oral delivery, which is subject to hepatic metabolic clearance and unpredictable absorption profiles (Baldwin et al., 2018). Additionally, intraperitoneal administration offered more consistent drug delivery, ensuring greater reliability of the experimental outcomes. This dosing regimen has been validated in several studies. According to Shi et al., significant attenuation of MF was observed in spontaneously hypertensive rats following its administration (Shi et al., 2007). Huang et al. showed that this dose effectively relieved myocardial hypertrophy in this salt-sensitive rat model (Huang et al., 2017).

The HIF-1α pathway is critically involved in cellular responses to high-salt stress. High-salt stimulation activates HIF-1α, which subsequently promotes fibrosis in multiple tissues, including kidney fibrosis and vascular remodeling (Della Penna et al., 2014; Deng et al., 2024). In MF, sustained hypoxia induces the activation of HIF-1α, which in turn facilitates fibrotic progression (Distler et al., 2007). HIF-1α facilitates fibrosis by inducing matrix overproduction, pathological neovascularization, and vascular alterations, thereby intensifying hypoxia and amplifying fibrotic damage (Xiong and Liu, 2017). High-salt stimulation significantly upregulated HIF-1α protein expression in both myocardial tissues of Dahl rats and cardiac fibroblasts. This upregulation can be attributed to the formation of a hypoxic microenvironment in the myocardial tissue induced by high salt intake. Several mechanisms contribute to hypoxia, including (1) damage to the coronary microcirculation, (2) oxidative stress enhancement, and (3) mitochondrial dysfunction (Mayyas et al., 2017; Oloyo et al., 2019; Sukumaran et al., 2020). Moreover, elevated reactive oxygen species (ROS) under high-salt conditions inhibit PHD activity, stabilizing HIF-1α by preventing its ubiquitin-mediated degradation (Chen et al., 2018; Liang et al., 2021). Additionally, high-salt intake suppresses PHD-2 expression, thereby inhibiting HIF-1α degradation and promoting its accumulation (Dallatu et al., 2013). Our findings showed that H_2_S supplementation markedly decreased HIF-1α protein levels in both Dahl rat myocardial tissues and primary cardiac fibroblasts, without altering *Hif1a* mRNA expression. This suggested that H_2_S acted at the post-translational level to suppress HIF-1α accumulation. These findings align with earlier studies demonstrating that H_2_S modulates HIF-1α through post-translational mechanisms. In particular, Dey et al. demonstrated that, under normoxic conditions, CBS-generated H_2_S persulfidated PHD2, enhancing its prolyl hydroxylase activity and thereby promoting HIF-1α degradation (Dey et al., 2020). Furthermore, Wu et al. showed that H_2_S suppressed HIF-1α translation through enhanced eIF2α phosphorylation, without affecting its transcriptional regulation (Wu et al., 2012). Together, these molecular insights highlight how H_2_S modulates HIF-1α signaling, thereby contributing to its antifibrotic effects under high-salt conditions.

HIF-1α-driven glycolysis facilitates both the proliferative and phenotypic transformation of CFs, contributing to fibrosis progression (Yu and Xu, 2015; Wang et al., 2020). Glycolysis promoted collagen synthesis through three main mechanisms: (1) providing amino acid precursors such as glycine, (2) generating lactate to enhance prolyl hydroxylase activity for collagen hydroxylation, and (3) supplying energy for post-translational modifications and fibril assembly of collagen molecules (Nigdelioglu et al., 2016). Thus, we investigated whether H_2_S modulated the expression of critical glycolytic enzymes, including GCK, LDHA and PFKM, which are direct targets of HIF-1α. RT-qPCR revealed markedly increased expression of *Gck, Ldha, and Pfkm* mRNA levels in cardiac fibroblasts cultured in high-salt medium compared to controls. However, NaHS treatment markedly suppressed transcription of these glycolysis-related genes. These results suggested that H_2_S inhibitd MF by downregulating HIF-1α and its transcriptional target glycolytic genes.

Furthermore, we utilized a HIF-1α stabilizer to examine the exact association between H_2_S-suppressed HIF-1α and the blocking impact of H_2_S on the cardiac fibroblast activation and MF induced by high salt stimulation. DMOG inhibited prolyl hydroxylase. It allows the HIF-1α protein to stabilize and accumulate within the nucleus (Shi et al., 2021). In our investigation, pre-treatment with DMOG antagonized the decrease in HIF-1α protein level induced by H_2_S and then reversed the inhibitory impact of H_2_S on cardiac fibroblast activation and collagen deposition.

Our subsequent experiments assessed the impact of high-salt conditions and NaHS intervention on HIF-1α regulation and fibrotic activity in NIH3T3 cells. High-salt conditions also elevated HIF-1α and fibrosis-related proteins, including collagen III, PCNA, and α-SMA, in NIH3T3 fibroblasts. Notably, NaHS treatment significantly suppressed high-salt-induced upregulation of HIF-1α and fibrosis markers. These findings mirrored those in primary rat fibroblasts, reinforcing the involvement of HIF-1α in high-salt-induced fibrosis and supporting NaHS as a potential intervention. In primary cardiomyocytes, high-salt treatment also significantly upregulated HIF-1α expression, whereas NaHS treatment suppressed this effect. The consistency of these results across different cell types highlights a universal mechanism underlying high-salt-induced HIF-1α activation, which may drive metabolic reprogramming in both fibroblasts and cardiomyocytes. Emerging evidence has shed light on the molecular pathways through which high-salt diets drive cellular dysfunction. For instance, high-salt stimulation of cardiomyocytes leads to mitochondrial damage and metabolic reprogramming, thereby promoting a hypertrophic phenotype (Zhao et al., 2024). Similarly, Zhu et al. demonstrated that suppressing HIF-1α alleviated glucolipid metabolic dysfunction in H9c2 cells subjected to AngII, hypoxia, or HIF-1α overexpression (Zhu et al., 2019). Together, these results underscore the central involvement of HIF-1α in modulating cellular adaptation under high-salt and hypoxic conditions, as well as the potential of therapeutic interventions targeting HIF-1α signaling. In H9c2 cells, H_2_S has been reported to alleviate CoCl_2_-induced oxidative stress and mitochondrial dysfunction, thereby protecting cardiomyocytes from hypoxic injury (Yang et al., 2011). This further highlights the cytoprotective potential of H_2_S, suggesting its involvement in modulating cardiomyocyte responses through interaction with the HIF-1α signaling pathway.

Beyond pharmacological H_2_S donors like NaHS, certain dietary components may augment endogenous H_2_S production. Cruciferous vegetables (e.g., broccoli and kale) and Allium species (e.g., garlic and onion) contain sulfur-rich compounds (glucosinolates and alliin/allicin, respectively) that are metabolized to release H_2_S (Spezzini et al., 2024). Studies have associated a higher intake of these foods with improved cardiovascular outcomes, including reduced atherosclerosis, hypertrophy, and lower hypertension risk (Sun and Ku, 2006; Li et al., 2011; Bradley et al., 2016). Furthermore, experimental evidence demonstrates that garlic extract significantly attenuates ventricular fibrosis in high-salt-fed Dahl rats (Hara et al., 2013). These findings imply that sulfur-containing dietary compounds may offer a clinically applicable means of H_2_S delivery. This potential is supported by population-based studies reporting associations between such dietary components and improved cardiovascular outcomes (Sobenin et al., 2010; Ried et al., 2018).

## 5 Conclusion

Attenuation of the endogenous H_2_S/CBS pathway in myocardial tissue contributes to MF under high-salt conditions in Dahl rats. Moreover, H_2_S inhibits cardiac fibroblast activation and collagen deposition induced by high-salt stimulation, primarily by downregulating HIF-1α protein levels. These results provide mechanistic insights into high-salt-induced MF and suggest that therapeutic modulation of the H_2_S/CBS pathway and HIF-1α signaling may represent a viable treatment strategy.

## Data Availability

The original contributions presented in the study are included in the article/Supplementary Material, further inquiries can be directed to the corresponding authors.

## Abbreviations

CBS, cystathionine-β-synthase; CSE, cystathionine-γ-lyase; DMOG, dimethyloxalylglycine; ECM, extracellular matrix; FBS, fetal bovine serum; H_2_S, hydrogen sulfide; HIF-1α, hypoxia-inducible factor-1α; MPST, mercaptopyruvate sulfurtransferase; NaCl, sodium chloride; NaHS, sodium hydrosulfide.
