# Supplementary material for: Hydrogen sulfide alleviates high-salt-stimulated myocardial fibrosis through inhibiting hypoxia-inducible factor-1α
Source: Front Pharmacol. 2025 Jun 26;16:1502269. doi: 10.3389/fphar.2025.1502269 (PMC12240975; doi:10.3389/fphar.2025.1502269)
Supplement: Supplementary file 1 [file DataSheet1.pdf]

Supplementary Information

Hydrogen Sulfide Alleviates High-Salt-Stimulated Myocardial Fibrosis Through Inhibiting Hypoxia-Inducible Factor-1α

The name(s) of the author(s)

Qian Peng[1#], Pan Huang[1#], Boyang Lv[1], Chaoshu Tang[2], Hongfang Jin[1,3]*, Yaqian Huang[1]*

[1]*Department of Pediatrics, Children's Medical Center, Peking University First Hospital, Beijing, China,*

[2]*Department of Physiology and Pathophysiology, Peking University Health Science Center, Beijing, China,*

[3]*State Key Laboratory of Vascular Homeostasis and Remodeling, Peking University, Beijing, China*

*CORRESPONDENCE:

Corresponding Author

Hongfang Jin,

jinhongfang51@126.com

Yaqian Huang,

yaqianhuang@126.com

Supplementary Table 1. Primers used in RT-qPCR.

Primer	Forward	Reverse
Cbs	CTCCGGGAGAAGGGTTTTGA	CATGTTCCCGAGAGTCACCAT
Col1a1	CAGAGCACCATTTTCCAAAGCA	GGTACAGAGTCTCTTGCTTCCT
Col3a1	AGGGCAGGGAACAACTGATG	GGTCCCACATTGCACAAAGC
Acta2	AGATCAAGATTATTGCTCCTCC	GGAAGGTAGATAGAGAAGCCA
Pcna	GGCGTGAACCTACAGAGCAT	CACAGGAGATCACCACAGCA
Hif1a	AGCAATTCTCCAAGCCCTCC	TTCATCAGTGGTGGCAGTTG
Gck	ATATTTTTTTAGGATTTGCC	ATATCACTCTCTTCGCGAAT
Ldha	CGTCTGCCCTATCAACTTTCG	CTTGGATGTGGTAGCCGTTTC
Pfkm	ATCACAGCCGAGGAGGCTAC	GGCGGCCCATCACTTCTAAC
Actb	TGTGCTATGTTGCCCTAGACTTC	ATTGCCGATAGTGATGACCTGAC

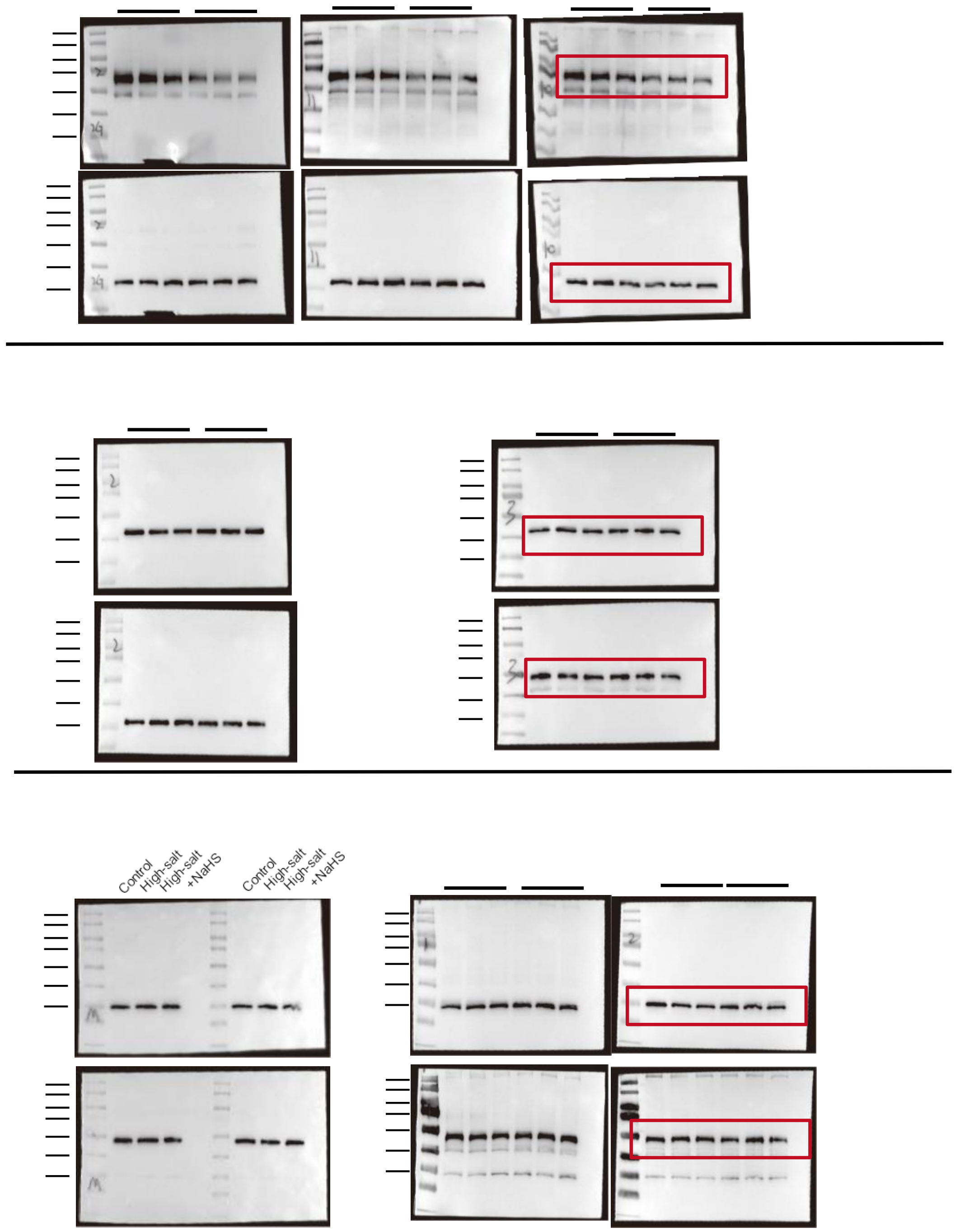

Supplementary Figure 1. Original unmodified blots used for Figures 1B and 1D.

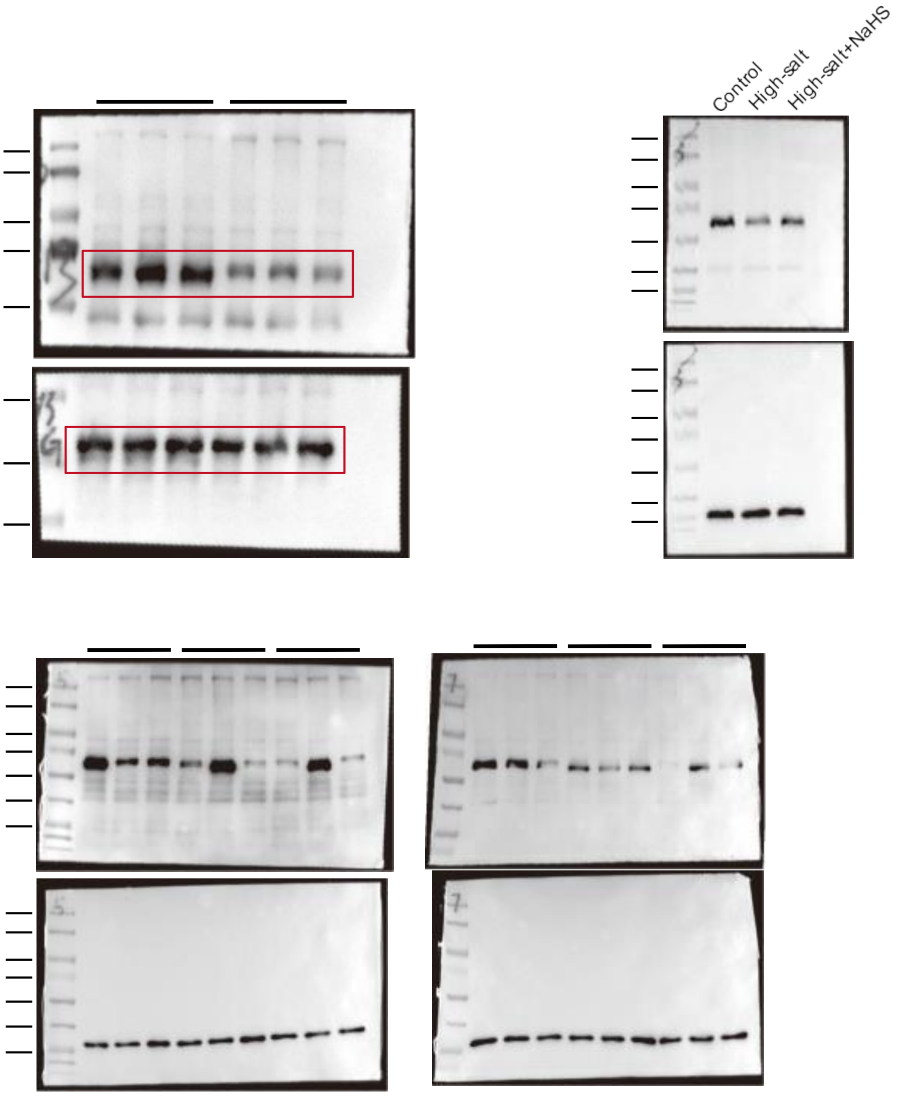

Supplementary Figure 2. Original unmodified blots used for Figure 1E.

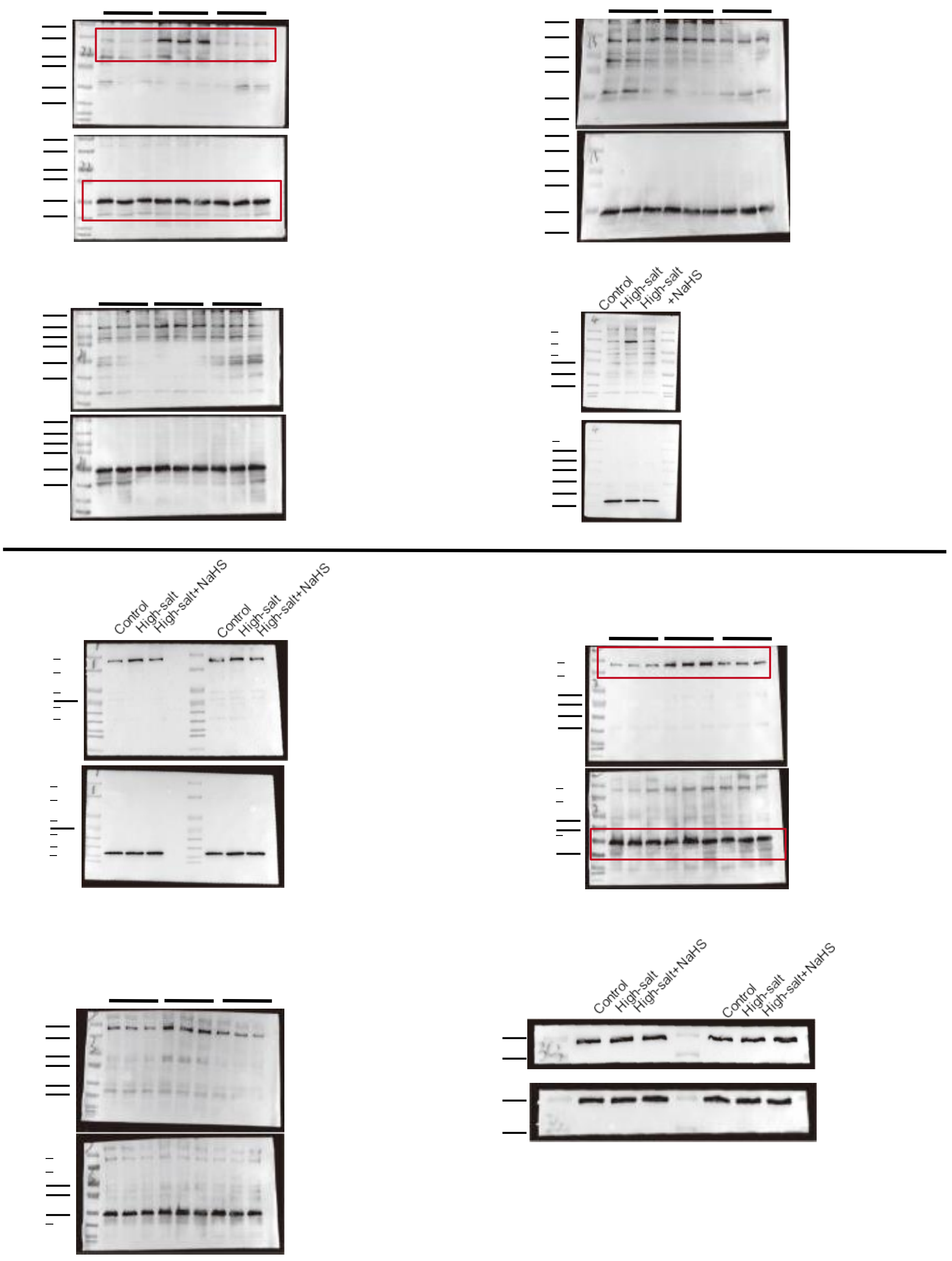

Supplementary Figure 3. Original unmodified blots used for Figure 2D.

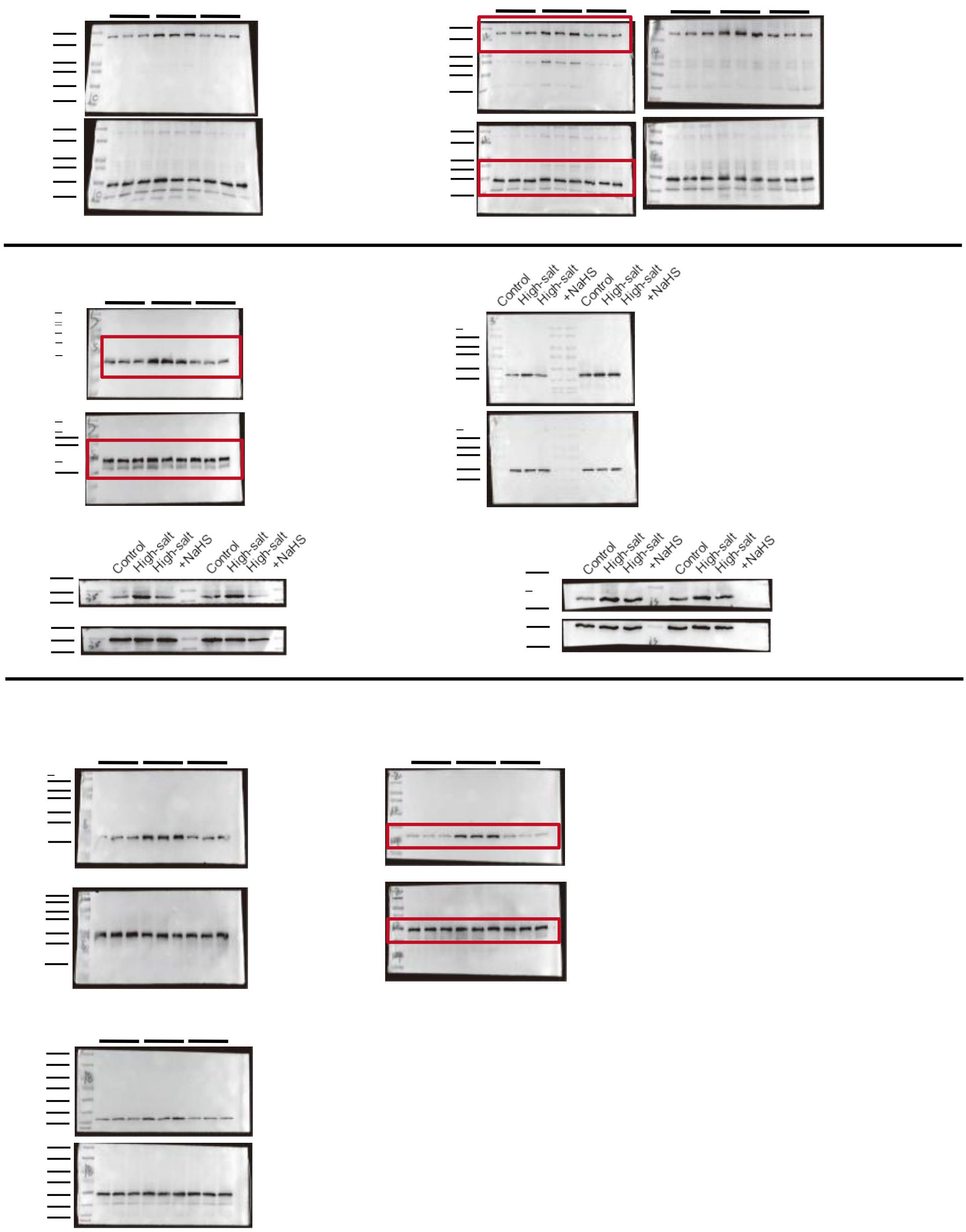

Supplementary Figure 4. Original unmodified blots used for Figure 3A.

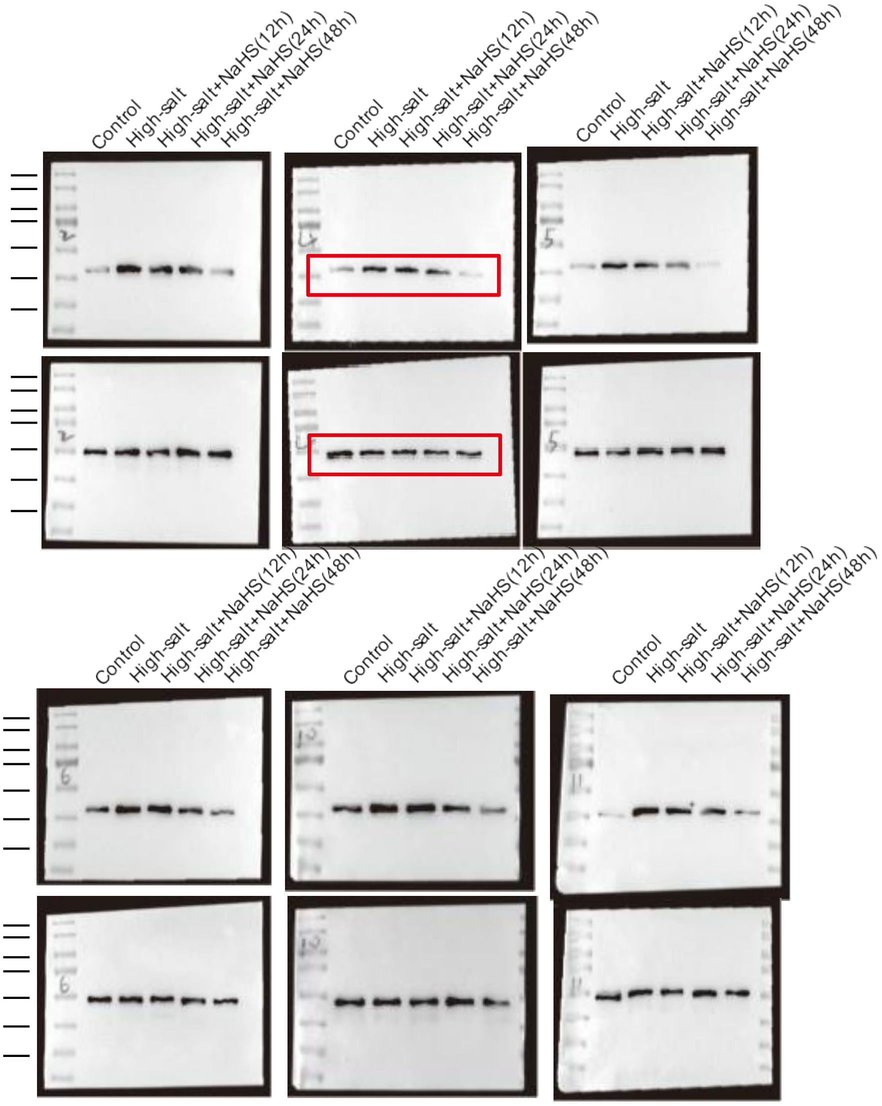
Control
High-salt
High-salt+NaHS(12h)
High-salt+NaHS(24h)
High-salt+NaHS(48h)

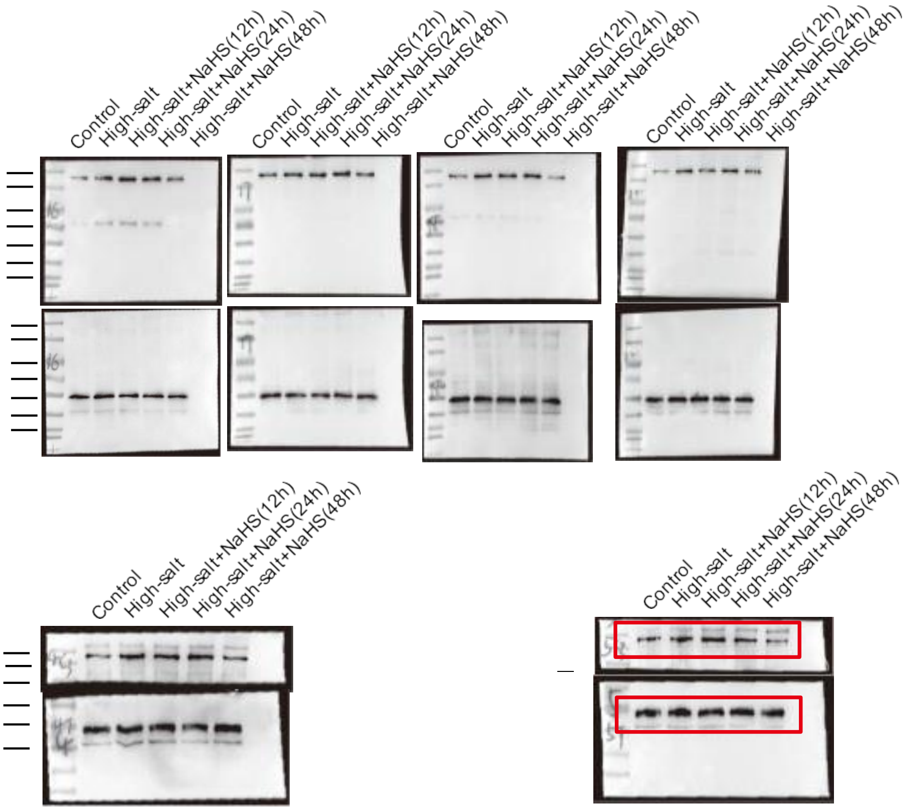

Supplementary Figure 5. Original unmodified blots used for Figure 3C.

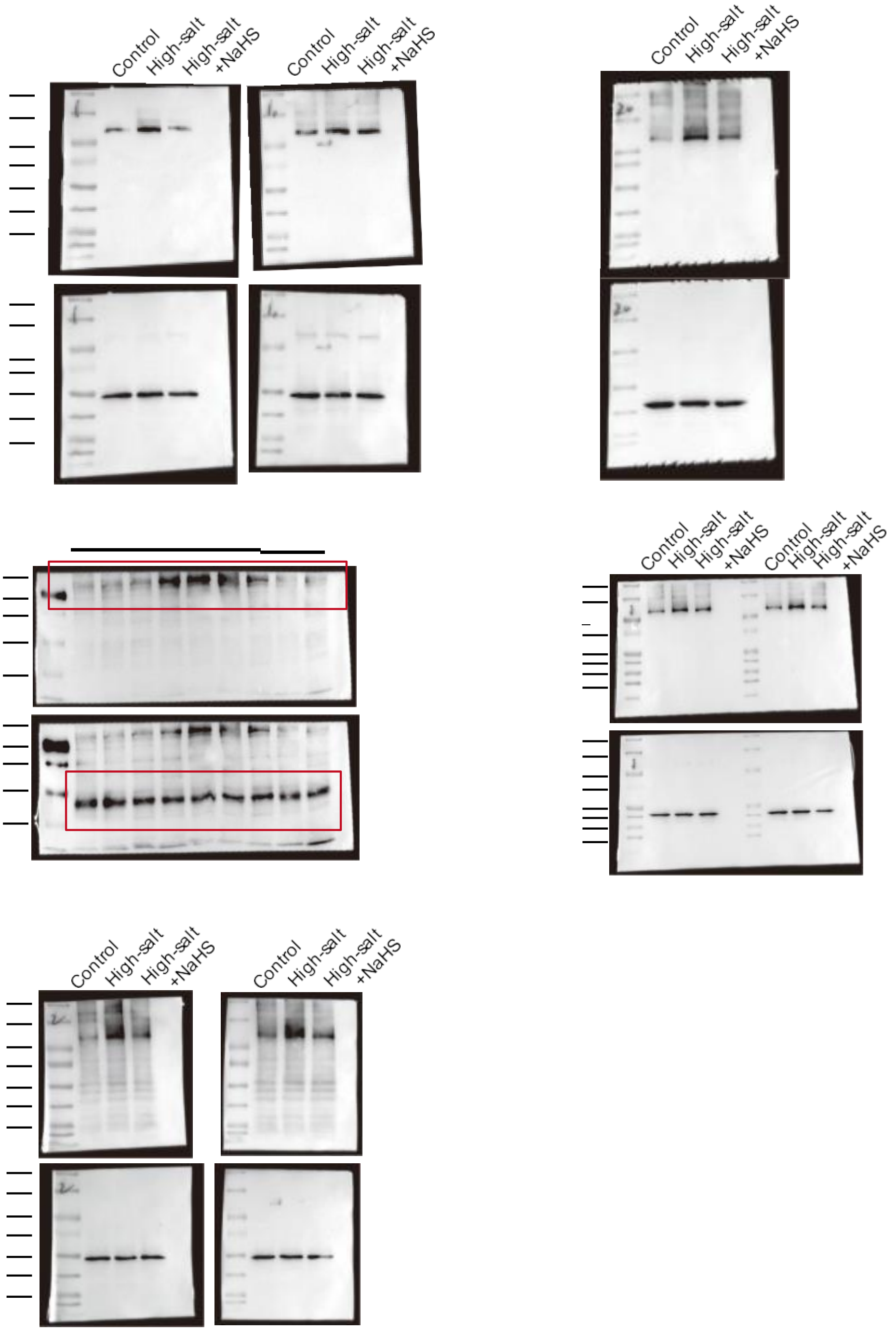

Supplementary Figure 6. Original unmodified blots used for Figure 3D.

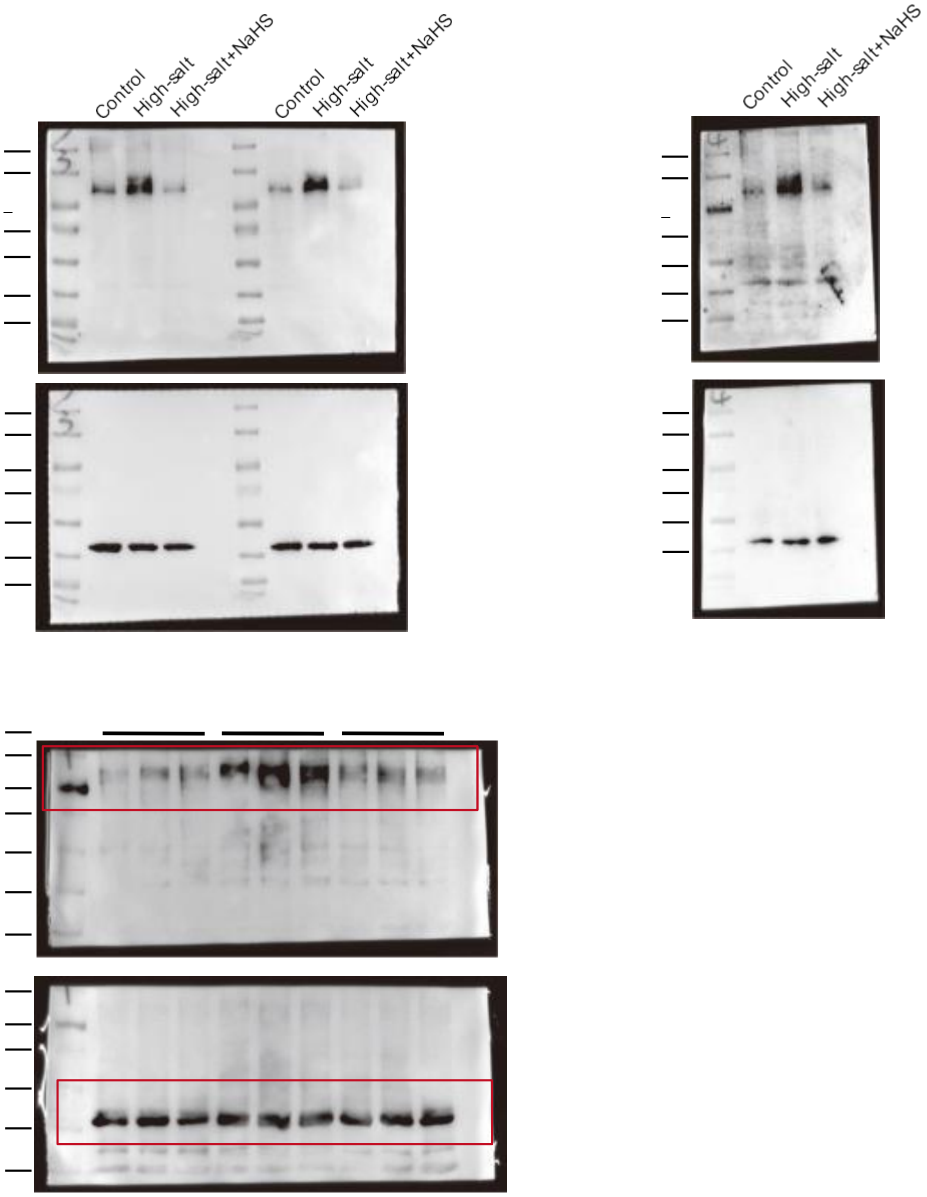

Supplementary Figure 7. Original unmodified blots used for Figure 3E.

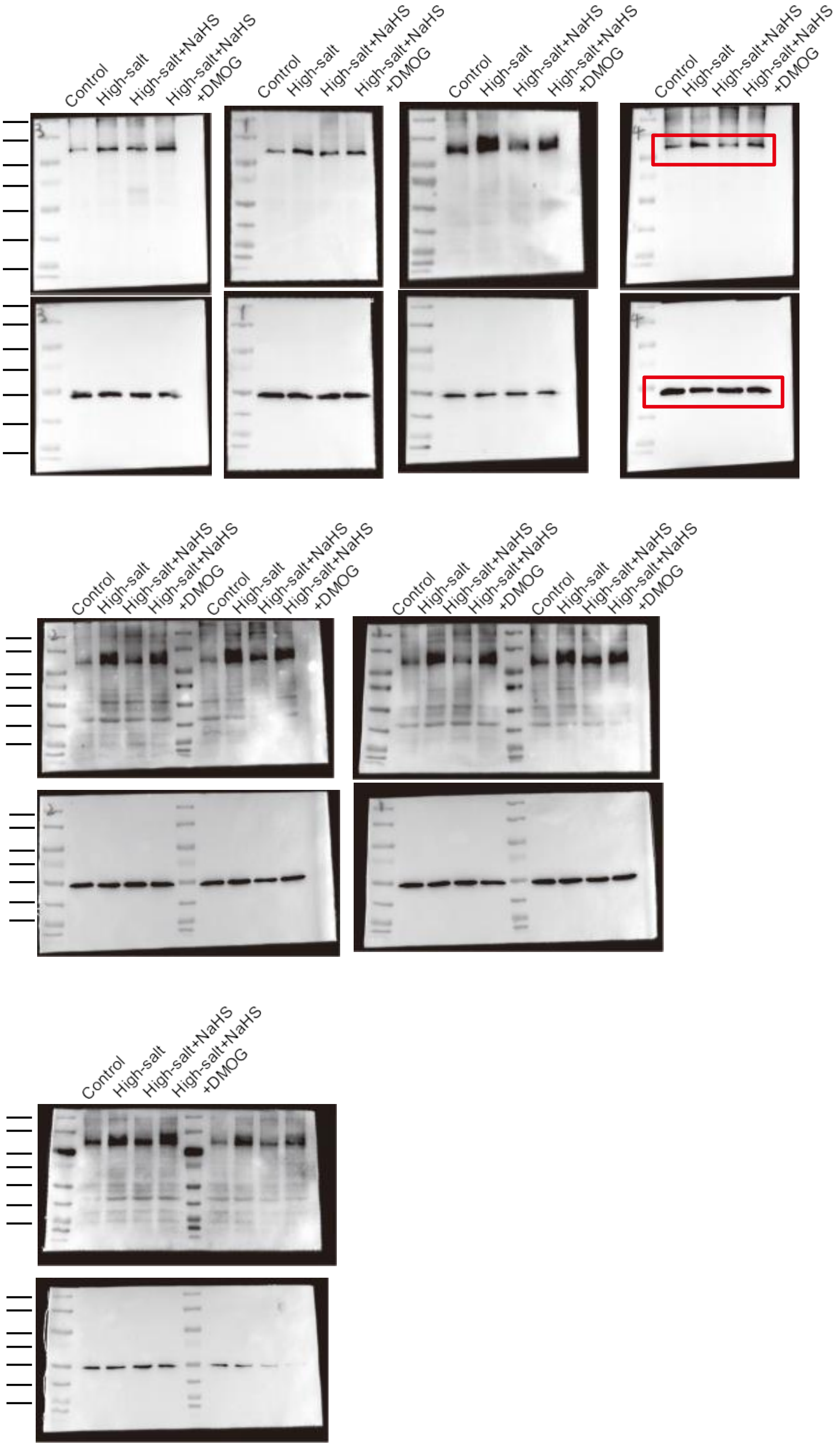

Supplementary Figure 8. Original unmodified blots used for Figure 4A.

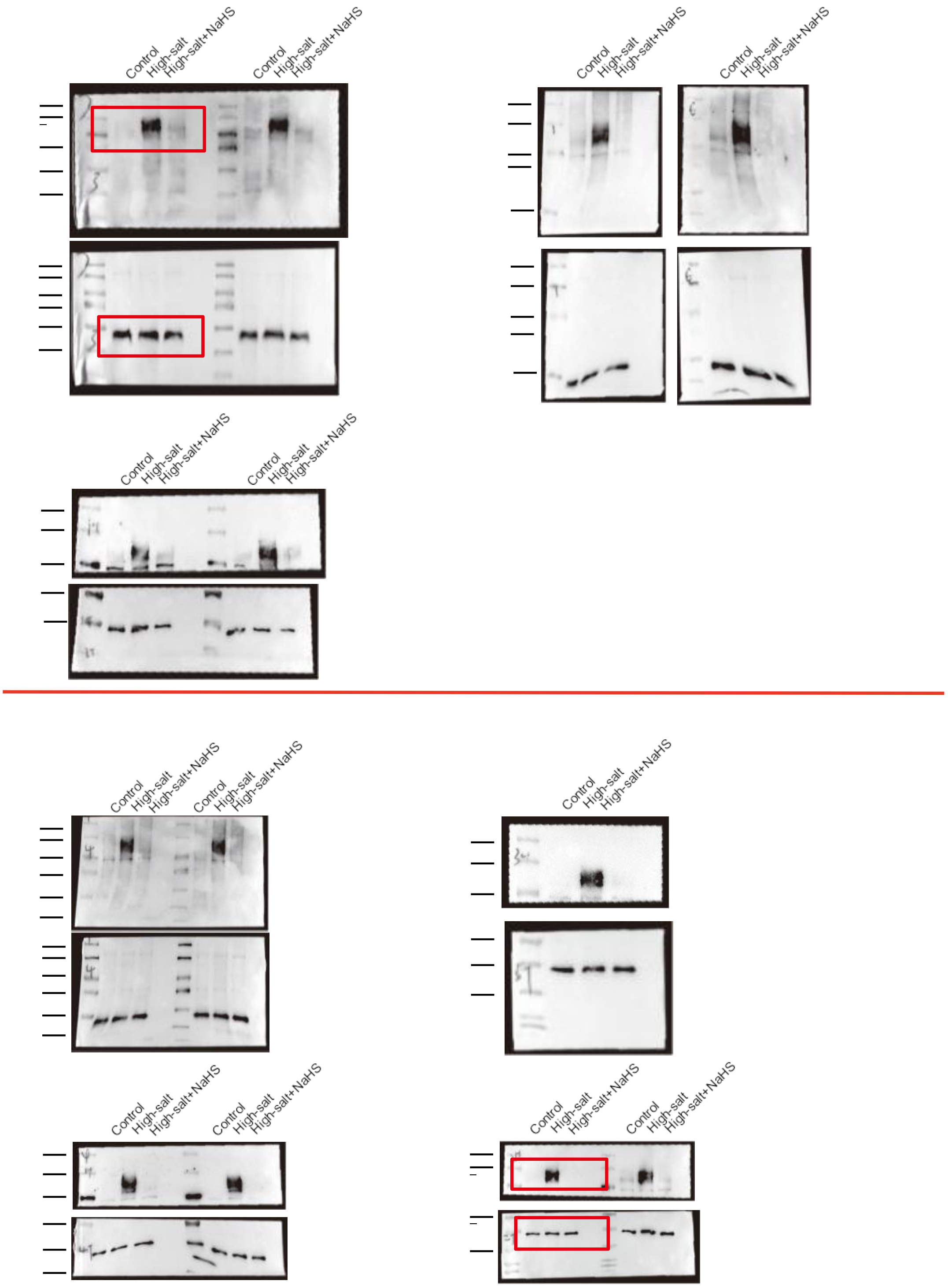

Supplementary Figure 9. Original unmodified blots used for Figures 5A and 5C.

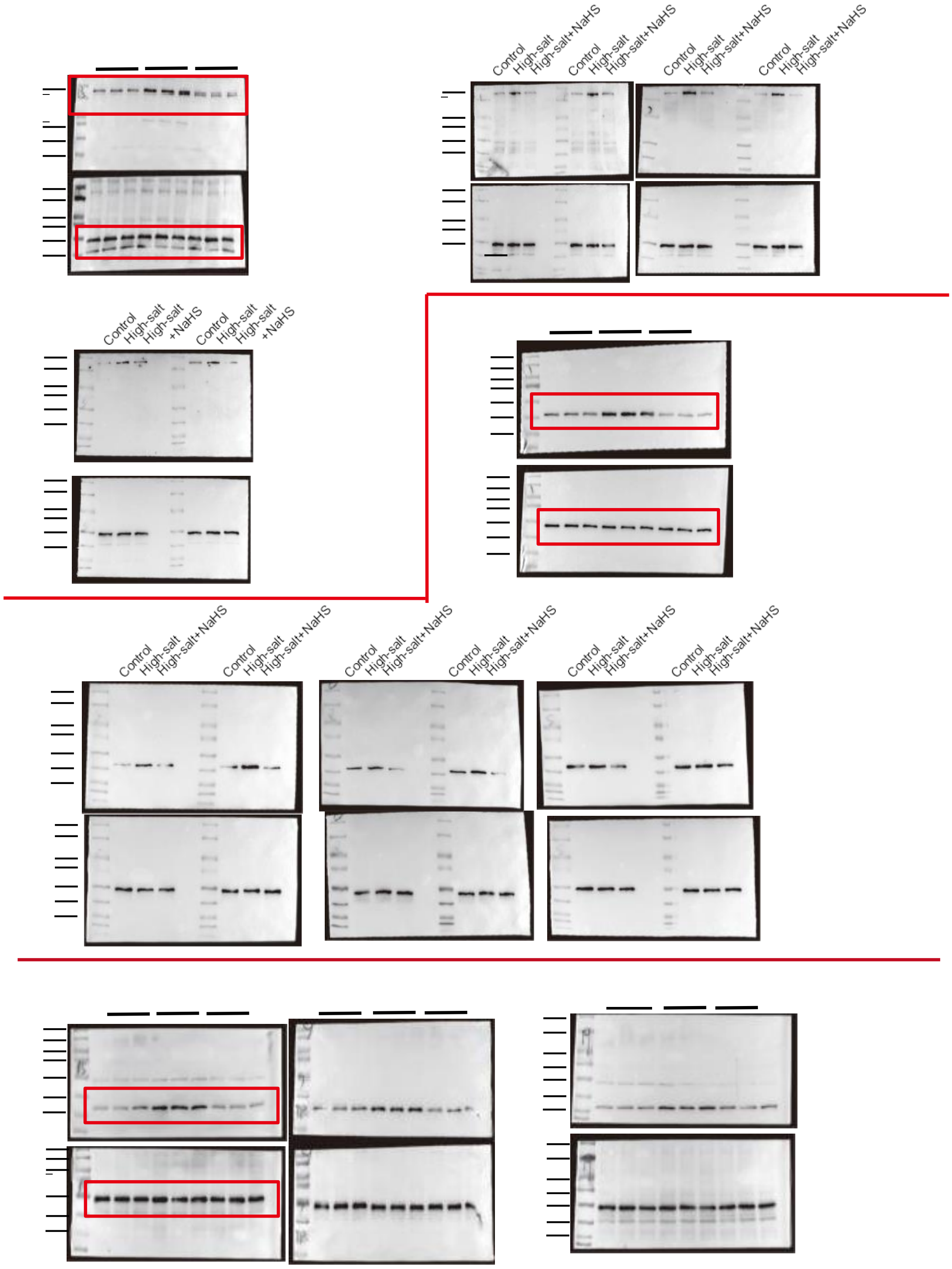

Supplementary Figure 10. Original unmodified blots used for Figure 5B.
